# Ethnic Disparities in Major Adverse Cardiac and Cerebrovascular Events (MACCEs) and Postoperative Outcomes Following Coronary Artery Bypass in Northeastern Iran (2007–2016)

**DOI:** 10.34172/aim.2023.81

**Published:** 2023-10-01

**Authors:** Mahin Nomali, Aryan Ayati, Amirhossein Tayebi, Keyvan Moghaddam, Soheil Mosallami, Gholamali Riahinokandeh, Mahdis Nomali, Gholamreza Roshandel

**Affiliations:** ^1^Department of Epidemiology and Biostatistics, School of Public Health, Tehran University of Medical Sciences, Tehran, Iran; ^2^Student Research Committee, Golestan University of Medical Sciences, Gorgan, Iran; ^3^Tehran Heart Center, Cardiovascular Diseases Research Institute, Tehran University of Medical Sciences, Tehran, Iran; ^4^Cardiovascular Research Center, Alborz University of Medical Sciences, Karaj, Iran; ^5^Supervisory Department, Kordkuy Amiralmomenin Hospital, Golestan University of Medical Sciences, Gorgan, Iran; ^6^Open Heart Intensive Care Unit, Kordkuy Amiralmomenin Hospital, Golestan University of Medical Sciences, Gorgan, Iran; ^7^Department of Surgery, School of Medicine, Sayyad Shirazi Hospital, Kordkuy Amiralmomenin Hospital, Golestan University of Medical Sciences, Gorgan, Iran; ^8^Shafa Heart Subspecialty Hospital, Golestan, Gorgan, Iran; ^9^Golestan Research Center of Gastroenterology and Hepatology, Golestan University of Medical Sciences, Gorgan, Iran

**Keywords:** Coronary artery bypass, Ethnicity, Iran, Major adverse cardiovascular events, Outcomes, Retrospective studies

## Abstract

**Background:**

Turkmens are an ethnic group mainly living in northeastern Iran. Despite previous studies on coronary artery bypass surgery (CABG) outcomes among different ethnicities, the effect of Turkmen ethnicity on outcomes of CABG surgery is still unknown. We aimed to assess the association between Turkmen ethnicity and postoperative outcomes following CABG.

**Methods:**

We used the CABG data from two heart centers in northeastern Iran between 2007 and 2016. We included adult patients undergoing CABG surgery. The study outcomes were in-hospital major adverse cardiac and cerebrovascular events (MACCEs), consisting of myocardial infarction (MI), stroke, and cardiovascular death, and postoperative outcomes, including postoperative arrhythmia, acute atrial fibrillation (AF), major bleeding, and acute renal failure (ARF).

**Results:**

Over the course of one decade, 3632 patients, with an average age (standard deviation) of 59.0 (9.8) years, were studied. Of these, 3,331 patients were of non-Turkmen ethnicity, and 301 patients were Turkmens. According to adjusted analysis, ethnicity was not associated with MACCEs (OR: 1.15, 95 % CI: 0.61, 2.16; *P*=0.663), postoperative arrhythmia (OR: 1.10, 95% CI: 0.78, 1.54; *P*=0.588), acute AF (OR: 1.17, 95 % CI: 0.83, 1.66; *P*=0.359), major bleeding (OR: 1.21, 95 % CI: 0.55, 2.67; *P*=0.636), or ARF (OR: 2.60, 95 % CI: 0.60, 11.75, *P*=0.224).

**Conclusion:**

This study found that despite ethnic disparity and preoperative differences, Turkmen ethnicity was not associated with in-hospital MACCEs, AF, major bleeding, or ARF after coronary artery bypass.

## Introduction

 Ischemic heart disease (IHD) is the major cause of death worldwide, responsible for about 9 million deaths in 2019. This number has increased by over 2 million since 2000, making it the most significant death increase due to any disease.^1^ Despite the growing use of percutaneous coronary interventions (PCIs), coronary artery bypass surgery (CABG) remains the most advantageous method for left main artery and multi-arterial revascularization.^2^

 Based on extensive studies conducted in past decades, risk factors and, subsequently, highly effective interventions have been introduced to lower the burden of IHDs.^3^ However, the majority of these studies were conducted in high-income countries, while recent data suggests that close to 80% of cardiovascular deaths occur in low- and middle-income regions.^4^ Furthermore, previous studies reported that ethnic disparities could significantly affect the frequency of risk factors and the severity of the IHDs.^5-7^

 Over half of the countries in the eastern Mediterranean region are considered low- or middle-income countries.^8^ As reported in the Eastern Mediterranean regional health observatory (EMRO), the disease burden in this region has shifted from communicable to non-communicable, expected to claim the greatest increase by 2030.^9-11^

 The Golestan province, located in northeastern Iran, is populated mainly by two ethnic groups, Fars and Turkmen. Previous studies have revealed different epidemiological patterns of various diseases and risk factors in these ethnic groups.^12-14^ The differences in cardiovascular risk factors include higher prevalence of metabolic syndrome,^15^ lower rate of obesity,^16-19^ and higher frequency of opium consumption in Turkmens compared to the other ethnicities.^20^ The exact reason for these differences is unknown; however, higher rural occupancy, genetic predisposition, and lifestyle variations are considered probable causes of inequality in cardiovascular disease (CVD) risk factors and severity.

 Although previous studies have assessed the effect of different ethnicities on CABG outcomes, the association between Turkmen ethnicity and CABG outcomes is still unknown. Enhancing the current understanding of this association is essential to develop better treatment strategies in this region. Therefore, this study evaluated the association between Turkmen ethnicity and postoperative outcomes following coronary artery bypass in northeastern Iran.

## Materials and Methods

###  Study Design, Setting, and Population 

 In this retrospective cohort study (REC ID IR.GOUMS.REC.1395.137), we used the data on patients undergoing CABG surgery between 2007 and 2016 in the Golestan province (northeastern Iran). Data related to two heart centers were acquired, including the Kordkuy heart center, affiliated with Golestan University of Medical Sciences (Gorgan, Iran), and Shafa Heart subspecialty hospital, the only centers providing heart surgeries to patients throughout the province.^21^ These centers utilize similar surgical techniques, pre-, peri-, and post-operative care for CABG patients. Therefore, patients older than 18 undergoing isolated on-pump CABG surgeries with distinguished ethnicity and outcome status were included in the analysis.

###  Operative Procedure 

 CABG surgeries were conducted for all patients according to the latest guidelines. After administering general anesthesia, a median sternotomy was performed, and the aorta and right atrium were cannulated to establish a cardiopulmonary bypass (CPB). A cardioplegic arrest was conducted utilizing St. Thomas crystalloid cardioplegic solution, and revascularization was performed during aortic cross-clamp.^22,23^ After the distal coronary anastomoses were completed, the aortic cross-clamp was removed, the heart was re-perfused, and the proximal anastomoses were completed using a partial occlusion clamp.^23^ Bypass vessels commonly used were the left internal thoracic artery and the greater saphenous vein.^24^

###  Variables and Measurement

 Study variables were demographics, medical history, comorbidities, lipid profiles, preoperative drugs, and preoperative and operative characteristics. Ethnicity and individual habits, including opium consumption and smoking, were self-reported variables. We classified ethnicity in the Golestan province into two categories: Turkmen and non-Turkmen, representing the main ethnic groups residing in the area. Obesity was defined as BMI equal to 30 and above, according to the Center for Disease Control and Prevention (CDC).^25^

 The study outcomes were in-hospital major adverse cardiac and cerebrovascular events (MACCEs) (i.e. MI, stroke, and cardiovascular death), postoperative arrhythmia, acute atrial fibrillation (AF), major bleeding, and acute renal failure (ARF). Further analysis regarding MACCE components is reported in Supplementary file 1.

- Myocardial infarction (MI) was established based on clinical evidence of acute myocardial ischemia, detection of an increase in cardiac troponin levels with at least one value over the 99th percentile upper limit, and at least one of the following: (1) Clinical symptoms of myocardial ischemia; (2) New ECG ischemia anomalies; (3) Formation of pathologic Q waves.^26^
- Stroke was diagnosed after imaging revealed the presence of a new focal neurological deficit lasting more than a day.^27^
- The attending cardiologists analyzed electrocardiograms to identify AF and arrhythmias following the operations.
- Major postoperative bleeding was considered as significant blood loss necessitating a second procedure.^28^
- ARF was diagnosed when there was a rise in plasma creatinine level of at least 0.3 mg/dL.^29,30^

###  Statistical Analysis

 First, the patients’ characteristics were compared based on their ethnicity. The normality assumption for continuous variables was evaluated graphically and statistically using the Shapiro-Wilk test. Continuous variables were reported as mean (standard deviation) or median (interquartile range) and compared using the independent sample t-test or the Mann-Whitney U test. Categorical variables were compared and reported as numbers and percentages using chi-square test. The association between Turkmen ethnicity and postoperative outcomes was determined using univariable logistic regression. We utilized the change-in-estimate (CIE) criterion with a 10% cutoff to identify potential confounding variables. Using multivariable logistic regression, we adjusted this association for potential confounding variables in accordance with the CIE. With a 95% confidence interval (CI), crude and adjusted odds ratios (ORs) were reported. The STATA/IC statistical software version 14.2 (StataCorp LP College Station, TX, USA) was used for data analysis.

## Results

**Figure 1:**
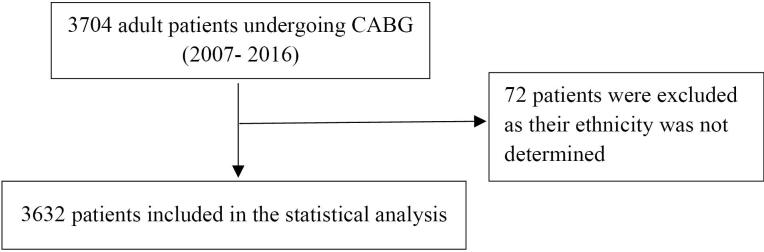
Study Population Diagram

 Between 2007 and 2016, we included 3,632 patients with an average (SD) age of 59.0 (9.8) (Figure 1). In this study, 3331 patients were of non-Turkmen ethnicity, and 301 patients were Turkmen (Table 1).

**Table 1:** Patients` Characteristics across the Ethnic Groups at Baseline

**Variables **	**Ethnic Group **		***P*** ** Value**
	**Non-Turkmen ** **(n=3331)**	**Turkmen ** **(n=301)**	
Age (y) [mean (SD)] (n = 3632)	59.0 (9.8)	60.0 (9.5)	0.922
< 65, n/N (%)	2388/3331 (72.0)	218/ 301 (72.0)	0.786
≥ 65, n/N (%)	943/3331 (28.0)	83/301 (28.0)	
Gender, n/N (%)			
Male	2097/3331 (63.0)	197/301 (65.0)	0.390
Female	1234/3331 (37.0)	104/301 (35.0)	
Obesity, n/N (%)			
Yes	918/3302 (28.0)	84/296 (28.0)	0.832
No	2384/3302 (72.0)	212/296 (72.0)	
Opium consumption, n/N (%)			
Ever users	905/3061 (30.0)	67/241 (28.0)	0.563
Never users	2156/3061 (70.0)	174/ 241 (72.0)	
Smoking, n/N (%)			
Ever users	421/3033 (14.0)	34/239 (14.0)	0.882
Never users	2612/3033 (86.0)	205/239 (86.0)	
**Medical History**			
Family history of cardiovascular diseases, n/N (%)			
Yes	2345/3273 (72.0)	207/286 (72.0)	0.792
No	928/3273 (28.0)	79/286 (28.0)	
Previous MI, n/N (%)			
Yes	186/3331 (6.0)	16/301 (5.0)	0.846
No	3145/3331 (94.0)	285/301 (95.0)	
**Comorbidities**			
DM, n/N (%)			
Yes	1568/3331 (47.0)	127/301 (42.0)	0.104
No	1763/3331 (53.0)	174/301 (58.0)	
Hyperlipidemia, n/N (%)			
Yes	1866/3288 (57.0)	181/282 (64.0)	0.015^*^
No	1422/ 3288 (43.0)	101/282 (36.0)	
HTN, n/N (%)			
Yes	1902/3243 (59.0)	153/273 (56.0)	0.402
No	1341/3243 (41.0)	120/273 (44.0)	
COPD, n/N (%)			
Yes	191/3210 (6.0)	15/260 (6.0)	0.905
No	3019/3210 (94.0)	245/260 (94.0)	
Lipid profile, Median (IQR)			
LDL (mg/dL)	146 (120-180)	149 (120-182)	0.491
Total cholesterol (mg/dL)	215 (181-271)	210 (166-271)	0.109
Non-HDL (mg/dL)	173.5 (138-234)	172 (115.5-229.5)	0.187
**Preoperative Medications **			
β-blockers, n/N (%)			
Yes	1549/3212 (48.0)	133/266 (50.0)	0.578
No	1663/3212 (52.0)	133/266 (50.0)	
Statins, n/N (%)			
Yes	2568/3319 (77.0)	235/297 (79.0)	0.488
No	751/3319 (23.0)	62/297 (21.0)	
Aspirin, n/N (%)			
Yes	2796/3319 (84.0)	258/297 (87.0)	0.231
No	523/3319 (16.0)	39/297 (13.0)	
**Preoperative Clinical Characteristics**			
LVEF (%), n/N (%)			
≤ 40 %	615/ 3300 (19.0)	77/294 (26.0)	0.002^*^
> 40 %	2685/3300 (81.0)	217/294 (74.0)	
Three vessel disease, n/N (%)			
Yes	2261/3151 (72.0)	205/284 (72.0)	0.878
No	890/ 3151 (28.0)	79/284 (28.0)	
LMCA stenosis, n/N (%)			
Yes	342/3176 (11.0)	40/258 (16.0)	0.020^*^
No	2834/3176 (89.0)	218/258 (84.0)	
**Operation Characteristics**			
Emergency CABG, n/N (%)			
Yes	152/3331 (5.0)	11/301 (4.0)	0.466
No	3179/3331 (95.0)	290/301 (96.0)	
LIMA graft, n/N (%)			
Yes	2970/3287 (90.0)	270/290 (93.0)	0.125
No	317/3287 (10.0)	20/290 (7.0)	
Number of grafts, n/N (%)			
≤ 3 grafts	1585/2998 (53.0)	138/275 (50.0)	0.393
> 3 grafts	1413/2998 (47.0)	137/275 (50.0)	
CPB time (minute) [median (IQR)] (n = 2215)	110 (58-130)	110 (55- 127)	0.449
Clamp time (minute) [median (IQR)] (n = 2213)	39 (30-50)	38 (28-45)	0.251

SD, standard deviation; IQR, interquartile range; MI, myocardial infarction; DM, Diabetes mellitus; HTN, hypertension; COPD, chronic obstructive pulmonary disease; AF, atrial fibrillation; LDL, low-density lipoprotein; ‎HDL, high-density lipoprotein; LVEF, left ventricular ejection fraction; LMCA, left main coronary artery; LIMA, left internal mammary artery; CPB, cardiopulmonary bypass. *Statistically significant (*P* value less than 0.05).

 The patients` characteristics across the ethnic groups at baseline are presented in Table 1. The mean age of patients, of Turkmen ethnicity or otherwise, was similar (*P* = 0.922). In both groups, the proportion of men was more than 60%. The proportion of obesity, opium consumption, smoking, family history of CVDs, previous MI, coexisting diabetes mellitus (DM), hypertension (HTN), chronic obstructive pulmonary disorder (COPD), preoperative drugs such as β-blockers, statins and aspirin, three-vessel disease, emergency CABG, left internal mammary artery (LIMA) graft and more than three grafts, CPB and clamp time were not different between the two groups. Despite no significant difference in lipid profiles between the two study groups, the low-density lipoprotein (LDL), total cholesterol, and high-density lipoprotein (HDL) cholesterol levels exceeded the normal ranges in both groups. However, the proportion of hyperlipidemia (*P* = 0.015), low left ventricular ejection fraction (LVEF) ( ≤ 40%) (*P* = 0.002), and left main coronary artery (LMCA) stenosis (*P* = 0.020) was higher among the patients with Turkmen ethnicity compared to non-Turkmen ethnicity (Table 1).

**Figure 2:**
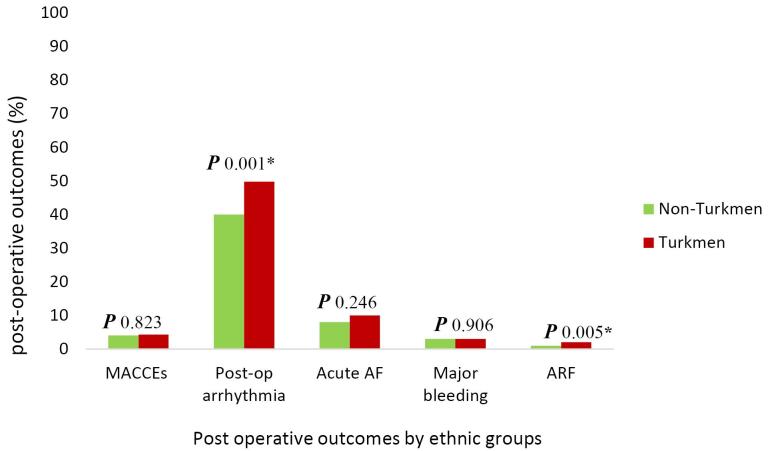
Postoperative Outcomes by Ethnic Groups.MACCEs, major adverse cardiac and cerebrovascular events (i.e., MI, stroke, and cardiovascular death); Post-op, Postoperative; AF, Atrial fibrillation; ARF, Acute renal failure; *P*, Probability value.^*^Statistically significant (*P* value less than 0.05).

Figure 2 shows the postoperative outcomes by ethnic groups. It represents a significant difference regarding postoperative arrhythmia (*P* = 0.001) and ARF between the two ethnic groups (*P* = 0.005).

Table 2 displays the in-hospital outcomes between the two ethnic groups, and crude and adjusted analysis results are also shown. In-hospital MACCEs were not different between the two groups (4% in non-Turkmen and 4.3% in the Turkmen ethnic group). Crude analysis indicated no association between ethnicity and in-hospital MACCEs (crude OR: 1.07, 95% CI: 0.60, 1.91; *P* = 0.823). Following adjustments by potential confounders, Turkmen ethnicity was not associated with major adverse events after CABG surgery (adjusted OR: 1.15, 95 % CI: 0.61, 2.16; *P* = 0.663) (Table 2).

**Table 2:** In-hospital Outcomes between Turkmen and Non-Turkmen Ethnic Groups Undergoing CABG Surgery

**In-hospital Outcomes **	**Ethnic Group**		**Crude OR** **(95% CI)**	***P*** **Value**^a^	**Adjusted OR** **(95% CI) **	***P*** **Value**^b^
	**Non-Turkmen (n=3331) **	**Turkmen (n=301) **				
MACCEs, n (%)			1.07 (0.60-1.91)	0.823	1.15 (0.61, 2.16)	0.663^c^
Yes	135 (4.0)	13 (4.3)				
No	3196 (96.0)	288 (95.7)				
Postoperative arrhythmia, n (%)			1.47 (1.15, 1.86)	0.001^a^	1.10 (0.78, 1.54)	0.588^d^
Yes	1344 (40.0)	150 (50.0)				
No	1987 (60.0)	151 (50.0)				
Acute AF, n (%)			1.26 (0.85, 1.88)	0.246	1.17 (0.83, 1.66)	0.359^d^
Yes	268 (8.0)	30 (10.0)				
No	3063 (92.0)	271 (90.0)				
Major bleeding, n (%)			0.96 (0.49, 1.85)	0.906	1.21 (0.55, 2.67)	0.636^e^
Yes	115 (3.0)	10 (3.0)				
No	3216 (97.0)	291 (97.0)				
Acute renal failure			3.74 (1.50, 9.50)	0.005	2.60 (0.60,11.75)	0.224^f^
Yes	18 (1.0)	6 (2.0)				
No	3313 (99.0)	295 (98.0)				

OR, odds ratio; CI, confidence interval; MACCE, major adverse cardiac and cerebrovascular events; AF, Atrial fibrillation. ^a^ Statistically significant (*P* < 0.2). ^b^ Statistically significant (*P* < 0.05). ^c^ Adjusted for age, sex, obesity, opium consumption, and smoking status. ^d^ Adjusted for opium consumption, smoking status, hyperlipidemia, hypertension, COPD, Beta-blockers, LIMA, CPB and clamp time. ^e^ Adjusted for family history of CVDs, hyperlipidemia, Beta-blockers, LMCA stenosis, CPB and clamp time. ^f^ Adjusted for CPB and clamp time.

 The proportion of postoperative arrhythmia was higher among patients with Turkmen ethnicity compared to non-Turkmen patients (49.8% vs. 40%, respectively). Crude analysis indicated that Turkmen ethnicity was significantly associated with arrhythmia (crude OR: 1.47, 95% CI: 1.15, 1.86; *P*= 0.001). In contrast, no association was found between ethnicity and postoperative arrhythmia after adjustment for potential confounders (adjusted OR: 1.10, 95% CI: 0.78, 1.54; *P* = 0.588) (Table 2). Even though Turkmen patients had a slightly higher percentage of acute AF compared to non-Turkmen patients (10% vs. 8%, respectively), the difference was not statistically significant (crude OR: 1.26, 95% CI: 0.85, 1.88; *P* = 0.246) and adjusted OR indicated no association (adjusted OR: 1.17, 95 % CI: 0.83, 1.66; *P* = 0.359) (Table 2). In addition, Turkmen and non-Turkmen patients experienced a similar proportion of major bleeding (3% in the two ethnic groups). According to crude analysis, there was no association between Turkmen ethnicity and major postoperative bleeding (crude OR: 0.96, 95 % CI: 0.49, 1.85; *P* = 0.906), and Turkmen ethnicity did not have any association with major bleeding (adjusted OR: 1.21, 95 % CI: 0.55, 2.67; *P* = 0.636). Although the ethnic group was associated with ARF in the univariable analysis (crude OR: 3.74, 95 % CI: 1.50, 9.50; *P* = 0.005), it was not a risk factor for ARF after adjustment for CPB and clamp time (adjusted OR: 2.60, 95% CI: 0.60,11.75, *P* = 0.224).

 Besides the variations in study settings and populations between the available literature, the definition of in-hospital MACEs differed between studies. Although we considered three components of MI, stroke, and cardiovascular death as in-hospital MACCEs, like most previous studies (Table 2), we assessed ARF as an additional component of in-hospital MACCEs (Supplementary file 1, Table S1). Therefore, there was no association between Turkmen ethnicity and in-hospital adverse events following CABG surgery, even after adding ARF as one of the MACCE components.

## Discussion

 Our retrospective cohort study compared 3331 non-Turkmen and 301 Turkmen patients undergoing CABG surgery. In both groups, most patients were under 65 years and were predominantly male. The history of hyperlipidemia was more common in Turkmen patients, but the history of DM, hypertension, and COPD was not significantly different between the two groups. Regarding the preoperative clinical characteristics, LMCA stenosis and lower LVEF were more prevalent among Turkmen patients. However, Turkmen ethnicity was not detected as a risk factor for in-hospital major adverse outcomes.

 Few studies have focused on the effect of Turkmen ethnicity on CVD risk factors and outcomes. In this regard, according to a study by Khoddam et al,^20^ Turkmen ethnicities are more likely to be uneducated, unemployed, and have lower income than non-Turkmen patients. These characteristics mirror their socioeconomic status and determine their sustainability for CVD. However, in their study, Turkmen ethnicity was not significantly associated with hypertension, physical activity level, obesity, and hyperlipidemia. In contrast, in our study, hyperlipidemia was more common among Turkmen patients. This inconsistency was probably due to the difference in the study population. As shown by Malekzadeh et al,^31^ lower wealth scores and socioeconomic status can lead to poorer knowledge of diseases, which may contribute to the disease occurrence.

 Our study revealed that more Turkmen patients had an LVEF less than 40% compared to non-Turkmen patients; also, LMCA stenosis was more common among these patients. This difference can result from a lower socioeconomic status, leading to worse and more difficult contact with health services, presenting with more severe and chronic diseases. Such a disparity was reported by Graham et al in the United States among Black, Hispanic, and non-Hispanic White patients suffering from acute MI.^32^ They reported that, on average, it takes longer for Black and Hispanic patients to receive surgical or medical treatments for MI. In this regard, Li et al assessed 207,570 patients admitted for acute MI in Pennsylvania from 1995 to 2006. They found that while 9.1% of Whites received CABG surgery within three months after MI, only 5.8% of Blacks underwent CABG within the same duration. This racial disparity was also reported in the PCI rates following MI (Whites vs. Blacks: 15.4% vs. 14.1%); the difference was more significant in counties with lower MI admission capacity.^33^

 Barnhart et al investigated the data of 12 555 MI patients hospitalized in New York City in 1996. They reported a significant difference among Whites, Hispanics, and Blacks regarding the baseline characteristics, medical history, and the chance of receiving PCI or CABG. However, the adjusted odds of in-hospital mortality were not significantly different when comparing Blacks or Hispanics with Whites. Although Blacks were less likely to receive revascularization, those who underwent PCI or CABG procedures had the same in-hospital outcome as Whites.^34^

 In our study, in-hospital adverse events following CABG surgery were reported in 4.3% and 4% of Turkmen and non-Turkmen patients, respectively. While it was thought that the ethnic disparity could affect the CABG outcome, our study showed that after adjusting for potential confounders, Turkmen ethnicity did not have any association with in-hospital MACCEs. Although postoperative arrhythmia and ARF were more common among Turkmen patients, our adjusted analyses showed no significant difference after confounder modification. Also, we detected no association between Turkmen ethnicity and postoperative acute AF or major bleeding.

 Although some researchers have studied CVD risk factors among Turkmen,^35,36^ no previous study has focused on the effect of Turkmen ethnicity on the outcomes of the CABG surgery. The northeastern region of Iran is notable for its large population of Turkmen ethnic groups.^37-40^ Understanding the underlying cause of ethnic disparities in this region requires a great effort to investigate patient characteristics, education, genetic predispositions, cultural norms, and the healthcare system. There is no easy way to study the interaction between these factors. Our study showed that surgical care for coronary artery disease is benefiting both Turkmen and non-Turkmen patients in northeastern Iran; however, the disparities will persist. We encourage future studies to focus on finding these differences and help further reduce them.

 In this large-scale study in northeastern Iran, in-hospital adverse events were evaluated among Turkmen ethnicity for the first time. Our study had some limitations. Because Turkmen and Fars ethnicities are the main ethnic groups, we could not compare our study results with other ethnicities in Iran. On the other hand, we only evaluated in-hospital outcomes. Therefore, additional longitudinal studies are needed to assess the adverse events following CABG between Turkmen and Non-Turkmen ethnicities.

## Conclusion

 To the best of our knowledge, this study is the first study in northeastern Iran to focus on the association between Turkmen ethnicity and in-hospital outcomes among patients undergoing CABG surgery. Turkmens undergoing CABG surgery were more likely to have hyperlipidemia and an LVEF less than 40%. Also, a higher percentage of Turkmens had LMCA stenosis post-surgery angiography. Despite the ethnic disparity and preoperative differences among the two ethnic groups, Turkmen ethnicity was not associated with in-hospital major adverse events, postoperative arrhythmia, acute AF, major bleeding, or ARF following on-pomp isolated CABG surgery.

## Supplementary Files

Supplementary file 1 contains Table S1.
