# Supplementary material for: Ethnic Disparities in Major Adverse Cardiac and Cerebrovascular Events (MACCEs) and Postoperative Outcomes Following Coronary Artery Bypass in Northeastern Iran (2007–2016)
Source: Arch Iran Med. 2023 Oct 1;26(10):554–60. doi: 10.34172/aim.2023.81 (PMC10862095; doi:10.34172/aim.2023.81)
Supplement: Supplementary file 1 — contains Table S1. [file aim-26-554-s001.pdf]

Arch Iran Med. October 2023;26(10):554-S1

10.34172/aim.2023.81

Supplementary file 1

Table S1. Comparison of In-hospital Major Adverse Events (+acute renal failure) across Ethnic Groups among Patients Undergoing Isolated on-pump CABG Surgery.

In- hospital outcomes	Ethnic group		Crude OR (95 % CI)	*P* value*	Adjusted OR (95% CI)	*P* value**
	Non-Turkmen (n=3331)	Turkmen (n=301)				
**MACCEs**, n (%)			1.42 (0.87, 2.32)	0.164	1.44 (0.71, 2.92)	0.313‡
Yes	151 (5.0)	19 (6.0)				
No	3180 (95.0)	282 (94.0)				

OR, Odds ratio; CI, Confidence Interval; *P* value, Probability value; MACCEs, major adverse cardiac and cerebrovascular events (i.e., MI, Stroke, cardiovascular death, and acute renal failure); AF, Atrial fibrillation.

*Statistically significant (P<0.2).

**Statistically significant (P< 0.05).

‡Adjusted for opium consumption, smoking status, number > 3 grafts, Cardiopulmonary bypass, and clamp time.
